# Elevated expression of Dickkopf-1 increases the sensitivity of human glioma cell line SHG_44 _to BCNU

**DOI:** 10.1186/1756-9966-29-131

**Published:** 2010-10-04

**Authors:** Youxin Zhou, Wenshuai Li, Qinian Xu, Yulun Huang

**Affiliations:** 1 Department of Neurosurgery, the First Affiliated Hospital of Soochow University, Suzhou 215006, China

## Abstract

**Background:**

Studies have shown that Dickkopf-1 (DKK-1) is involved in tumorigenesis. Recently, we found that 9 out of 12 human glioma cell lines had high level of DKK-1 protein while the other 3 had very low or non-detectable level of DKK-1. The aim of this study is to further examine the function of DKK-1 in glioma cells.

**Materials and methods:**

The glioma cell line SHG_44 _was obtained from a patient with grade II-III astrocytoma. SHG_44 _cells were transfected with a human DKK-1 gene. Transfection of the empty vector pcDNA3.1 was used as negative control. Sensitivity to BCNU was measured by Annexin-V staining. Expression of bax, bcl-2 and caspase-3 of three groups was determined by immunohistochemistry.

**Results:**

The tranfection was confirmed by PCR, RT-PCR and Western blot. More apoptotic cell death was observed in the DKK-1 transfected cells, comparing to the non-transfected cells, or cells with empty vector. The expression of bax and caspase-3 of the SHG_44 _-DDK-1 increased, whereas the expression of bcl-2 decreased

**Conclusion:**

Our results indicated that DKK-1 has a pro-apoptotic function of in glioma.

## Introduction

Dickkopf-1(DKK-1) gene was first discovered in 1998 as a head formation inducer and an antagonist of Wnt signaling pathway [1]. In normal tissues of human body, DKK-1 mRNA was highly expressed in placenta and at a very low level in prostate only [2,3]. Recent studies have revealed the involvement of DKK-1 protein in tumorigenesis. Its exact role in tumorigenesis, however, still remains unclear. Several studies reported that the expression level of DKK-1 in different tumors was different and its biological functions were different as well [4-8]. DDK-1 expression was confirmed in several cancer cell lines derived from breast and other common cancers. DDK-1 protein secretion was documented in breast, prostate and lung cancers, but was negligible in melanoma [9]. The DKK-1 concentration was significantly higher in the serum of lung cancer patients than in that of other malignant tumor patients or healthy people. The DKK-1 concentration is significantly lower in serum of patients with gastric cancer, colorectal cancer, ovarian cancer, and cervical adenocarcinoma compared with healthy people [10]. DKK-1 is a candidate gene for tumor suppressor in glioma and considered as a serologic and prognostic biomarker.In our recent study of 12 human glioma cell lines, we found that the supernatant fluid and lysate of 9 cell lines had high level of DKK-1 protein and the other 3 had very low level or non-detectable DKK-1 protein (Zhou et al, unpublished data). The high level of DKK-1 protein in most glioma cell lines suggested that DKK-1 may play an important role in glioma and attracted our intention to further study this DKK-1's function in glioma.

In this study we constructed a eukaryotic expression vector of human DKK-1(pcDNA3.1-DKK-1) and stably transfected the vector into the glioma cell line SHG_44_, which had no expression of DKK-1 under normal growth condition. We found that elevated expression of DKK-1 increased the sensitivity of SHG_44 _cells to the anti-cancer drug BCNU *in vitro*.

## Materials and methods

### Construction of expression vector

The 816-base pair human DKK-1 cDNA was amplified from the RNA of human placenta tissue using reverse transcription polymerase chain reaction (RT-PCR). The sequence of sense primer was 5'-CTAGCTAGCACATGATGGCT CTGG-3' (NHe I enzyme digestion site was indicated as underline) and antisense primer was 5'-GGAATTCGTGTCTCTGACAAGTGTG-3' (EcoR I enzyme digestion site was indicated as underline). The PCR reaction (10 μl) contained 1 μl cDNA, l μl 10 × buffer (MgCl_2_), 0.4 mM dNTPs, 1umol primer, 1U TaqDNA Polymerase. After denaturation at 95°C for 5 min, PCR was performed for 35 cycles (30 s at 95°C, 30 s at 50°C and 30 s at 72°C) and extended at 72°C for 5 min. The linear NHeI-EcoRI fragment containing the DKK-1 cDNA was subcloned into pcDNA3.1 (Invitrogen Company), which yielded pcDNA3.1-DKK-1 by T4 ligase (TaKaRa Company). The insertion of DDK-1 in pcDNA3.1 was confirmed by PCR, restriction enzyme digestion analysis (NHeI and EcoRI) and DNA sequencing.

### Cell culture

The human glioma cell line SHG_44 _was established by our lab in 1984 and has been widely used in China. It was originally obtained from a patient with grade II-III astrocytoma (according to World Health Organization). Cells were cultured in RPMI1640 medium (Giboc Company) supplemented with 10% fetal bovine serum, 100 IU/ml penicillin and 100 μg/ml streptomycin. Cells were cultured at 37°C in a humidified atmosphere containing 5% carbon dioxide. The culture medium was changed every 48 h.

### Determination of the optimal concentration of G418

G418 is an aminoglycoside and is commonly used as a selective agent for the bacterial neo r/kan r genes. The optimal concentration of G418 for selection of resistance was determined by the following procedure. SHG_44 _cells were plated at the same concentration of 5 × 10^4^/well, in 24-well plates containing 2 ml culture medium per well. G418 (sigma Company) was added to wells at 10 different concentrations (75, 100, 150, 200, 300, 400, 500, 600, 700, 800 μg/ml). The culture media were changed once per 48 h. The lowest G418 concentration, in which all cell died after 12-14 days culture, was chosen as the optimal concentration for resistance selection.

### Transfection of SHG_44 _cells with pcDNA3.1-DKK-1

For stable transfection of the DKK-1 gene, SHG_44 _cells (1 × 10^6 ^) were plated in 6-well plates 24 h before transfection. Lipofectamine 2000 (Invitrogen Company) was used to mediate transfection using 5.0 μg of pcDNA3.1-DKK-1 vector or 5.0 μg of empty pcDNA3.1 vector as a control according to the manufacture's protocol. After 48 h transfection, the cells were selected in media supplemented with G418 (150 μg/ml). The medium was changed once per 48 h. Non-transfected SHG_44 _cells died within two weeks. G418-resistant cells were selected and named as SHG_44_-DKK-1. Cells with empty vector of pcDNA3.1 were named as SHG_44_-EV.

### PCR confirmation of DKK-1 in SHG_44 _cells

DNA from cells of normal SHG_44_, SHG_44 _-EV, SHG_44_-DKK-1 was isolated using a DNA extraction kit (Puregenetm DNA isolation kit, Gentra systems). A portion of the DKK-1 gene was used to design the primers. The upstream primer sequence was 5'-TCACGCTATGTGCTGCCCCG-3' and downstream 5'-TGAGGCACAGTCTGATGACCGGA-3'. The expected product was 223 bp. PCR reaction system (50 μl) was: 3 μl cDNA, 5 μl 10 × Buffer, 4 μl MgC1_2, _1 μl dNTP, 1 μl primer, 0.3 μl TaqDNA Polymerase. PCR reaction condition was: an initial denaturation step of 94°C for 7 min, followed by 30 cycles of a three-step program of 94°C for 30 s, 56°C for 30 s, 72°C for 45 s, and a final extension step of 72°C for 7 min. All the products were electrophoresed on the agarose gel.

### RT-PCR of DKK-1 mRNA

Analysis of the DKK-1 mRNA expression of the three groups of cells (normal SHG_44_, SHG_44_-EV and SHG_44_-DKK-1) was performed by RT-PCR. Total RNA from cell lines was isolated using Trizol (Invitrogen Company). The purity and concentration of total RNA were detected by UV chromatogram analyzer (Backma Company). The concentration of RNA was adjusted to 1 μg/μl. β-actin was used as an internal control to ensure RNA quality and loading accuracy. Primer sequences were 5'-AGCGAGCATCCCCCA AAGTT-3' (upstream) and 5'-GGGCACGAA GGCTCATCATT-3' (downstream). The predicted product size is 285 bp. The primers for DKK-1 were the same mentioned above. The PCR condition for DKK-1 and β-actin was the same as described above.

### Western blot analysis

The total protein of the three groups of cells (normal SHG_44_, SHG_44_-EV, SHG_44_-DKK-1) was extracted directly in the lysis buffer and the concentration of total protein was quantified by UV chromatogram analyzer. 50 μg protein was separated using 12% sodium dodecyl sulfate- polyacrylamide gel (SDS-PAGE). After electrophoresis, proteins were transferred from gel to zapon fibrous membrane and the membrane was blocked by 5% non-fat milk. Monoclonal mouse anti-human DKK-1 antibody (R & D Company) (1:1000 dilution) was probed. For loading control, membranes were striped and reprobed with mouse anti-human β-actin antibody (R&D Company)(1:1000 dilution). Horseradish peroxidase (HRP)-conjugated goat-anti-mouse antibody (Coulter Immunotech Company) were added. Protein bands were visualized using the enhanced chemiluminescence system (Millipore Company).

### Apoptosis analysis

Normal SHG_44_, SHG_44._-EV and SHG_44_-DKK-1 cells were incubated in 6-well plates by 1 × 10^6 ^cells/well) in medium with or without 50 μM BCNU (Medical Isotopes Company) for 24 hours. Apoptosis was detected using the Annexin V-FITC Apoptosis Detection Kit (Jingmei Company). Briefly, cells were harvested and then resuspended in 1 ml of buffer followed by addition of 5 μl Annexin V and 10 μl PI. Cells were incubated in the dark at room temperature for 15 min. Cell death was determined using a flow cytometer (Backman Company). Data were obtained and analyzed by CellQuest software (Largo Company).

### Immunohistochemical analysis for bax, bcl-2 and caspase-3

Normal SHG_44_, SHG_44._-EV and SHG_44_-DKK-1 cells were incubated in 6-well plates by 1 × 10^6 ^cells/well in medium with or without 50 μM BCNU (Medical Isotopes Company) for 24 hours. Cells were washed in 0.05 M phosphate-buffered saline (PBS) (pH. 7.4) for 15 minutes then fixed in 4% paraformaldehyde for 20 minutes. Streptavidin/biotin-peroxidase (SP) method was used for immunohistochemical staining. The primary antibodies, namely antibodies against bax, bcl-2 and caspase-3 (Wuhan Boster Biological Technology, China), were diluted at 1:100. PBS was used as control. Labeled cells was photographed and the integrated optical density (IOD) was measured using Image pro plus 5.02 (Media Cybernetics, USA).

### Statistical analyses

The difference between controls and treated groups were analyzed by χ^2^-test. Differences were considered significant if P < 0.05. Statistics was performed with SPSS 13.0 software for Windows (LEAD Technologies, Chicago, IL, USA).

## Results

### DKK-1 cDNA amplification and identification of expression vector

We first designed the primers and amplified the 816 bp DKK-1 gene from human placenta tissue. The PCR product was collected and purified. The purified DKK-1 fragment and pcDNA3.1 vector were digested by NHe I and EcoR I, followed by ligation with T4 ligase at 16°C for overnight. The ligated plasmid was transformed into DH5α strain of E. coli. Single colonies were selected and PCR amplification confirmed a single band of 816 bp. The plasmids were isolated from DH5α and digested by NHe I and EcoR I. DNA gel showed two bands, one corresponding to the 816 bp fragment and the second one corresponding to the vector pcDNA3.1. DNA Sequencing showed that the 816 bp fragment matched with the DNA sequence of DKK-1 gene.

### Cell morphology and SHG_44_-DKK-1 screening

**Figure 1:**
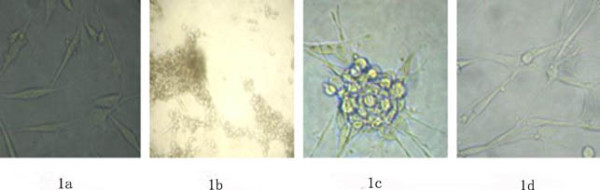
**Microscopic images of different groups cells in selection**. Normal SHG_44 _(1a), normal SHG_44 _cells cultured in the presence of G418 for two weeks (1b); and SHG_44_-DKK-1 cells cultured in the presence of G418 for three weeks (1c, 1d).

Normal SHG_44 _cells were usually elongated and football shaped (Fig.1a). They died within two weeks when cultured in the presence of 150 μg/ml G418 (Fig.1b). Cells transfected with pcDNA3.1-DKK-1 were resistant to G418 and were screened under G418 for about three weeks and named as SHG_44_-DKK-1. The SHG_44_-DKK-1 cells appeared similar to the non-transfected cells and sometimes formed clusters (Fig.1c, d).

### PCR analysis of DKK-1 in SHG_44 _cells

**Figure 2:**
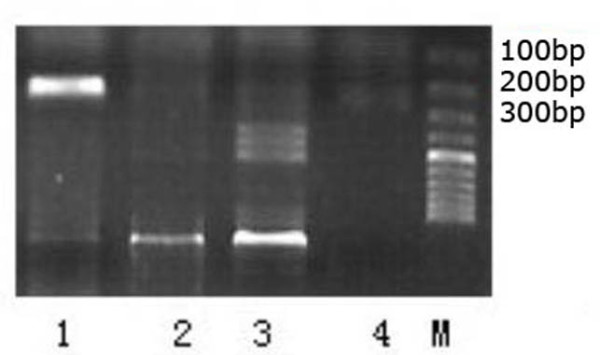
**PCR amplification of DKK-1 SHG_44_-DKK-1 cells was lane 1, SHG_44_-EV was lane 2, normal SHG_44 _cells was lane 3 and control (culture medium only) was lane 4**. M was the marker for standard DNA molecular mass.

DNA was extracted from cells of normal SHG_44, _SHG_44_-EV and SHG_44 _-DKK-1. The extracted DNA was amplified by PCR using the primer pair described above. As expected, a 223bp fragment was observed in SHG_44 _-DKK-1cells, but not in normal SHG_44, _or SHG_44 _-EV cells (Fig.2). This result further confirmed the specific transfection of DKK-1 gene into the SHG_44 _cells.

### DKK-1 mRNA expression in SHG_44 _cells

**Figure 3:**
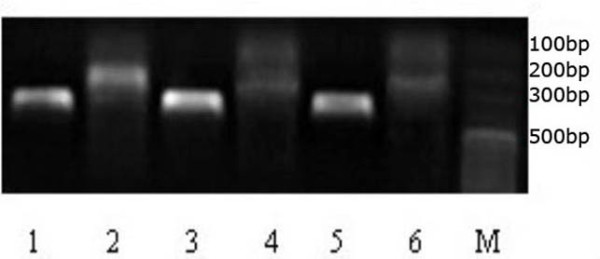
**RT-PCR analysis of DKK-1 mRNA expression**. Lane 1, 3 and 5 β-actin from cells of SHG_44_-DKK-1, SHG_44_-EV and normal SHG_44 _respectively. Lane 2, 4, 6 were DKK-1 mRNA from cells of SHG_44_-DKK-1, SHG_44_-EV and normal SHG_44 _respectively. M was the marker of standard DNA molecular mass.

RNA extracted from normal SHG_44, _SHG_44_-EV and SHG_44 _-DDK-1 cells was amplified by RT-PCR and subsequently analyzed by DNA gel. A prominent 223 bp band was detected from SHG_44 _-DKK-1 cells, but non-detectable from SHG_44 _-EV cells or normal SHG_44 _cells (Fig.3).

### DKK-1 protein expression in SHG_44 _cells

**Figure 4:**
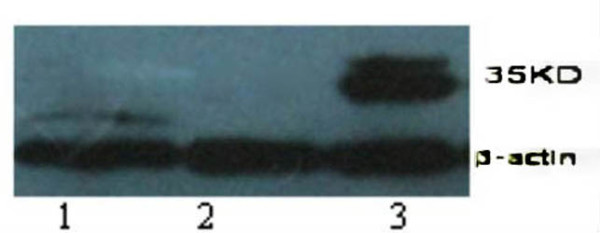
**Western blot analysis of DKK-1 protein**. It showing DKK-1 protein from cells of normal SHG_44 _(lane 1), SHG_44_-EV (lane 2) and SHG_44_-DKK-1 (Lane 3). β-actin was used as loading control.

The total protein exacted from normal SHG_44_, SHG_44_-EV and SHG_44 _-DDK-1 cells was separated using 12% SDS-PAGE and was subsequently analyzed by Western blot. A 35KD band, which corresponds to the size of DKK-1 protein was observed in SHG_44 _-DKK-1 cells, but not in SHG_44 _-EV or normal SHG_44 _cells (Fig.4).

### BCNU induced apoptosis

**Figure 5:**
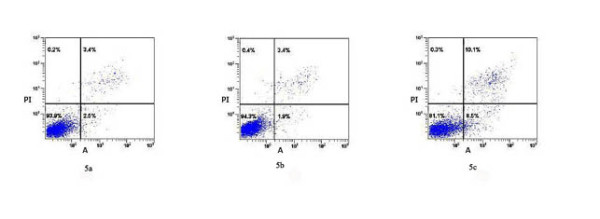
**Apoptosis ratio was detected by flow cytometry analysis**. Representative image of flow cytometry analysis of BCNU treated cells, showing the apoptosis ratio (right lower-quadrant) of normal SHG_44 _(a), SHG_44_-EV (b) and SHG_44_-DKK-1 (c) cells.

BCNU is an anti-cancer drug and an inducer of apoptotic cell death. We used BCNU to further assess the role of DKK-1 in SHG_44 _cells. Apoptosis was observed in all three groups of cells: normal SHG_44_, SHG_44_-EV and SHG_44 _-DDK-1. The average apoptosis ratio of normal SHG_44 _, SHG_44_-EV cells and SHG_44 _-DKK-1, was2.5 ± 0.2%, 1.8 ± 0.2%, 8.4 ± 0.3%, respectively(Fig.5). The apoptosis ratio of SHG_44 _-DKK-1 cells was significantly (P < 0.05) higher than that of normal SHG_44 _or SHG_44_-EVcells. Minimal apoptosis was observed in all three groups of cells in the absence of BCNU.

### Immunohistochemistry analysis

**Figure 6:**
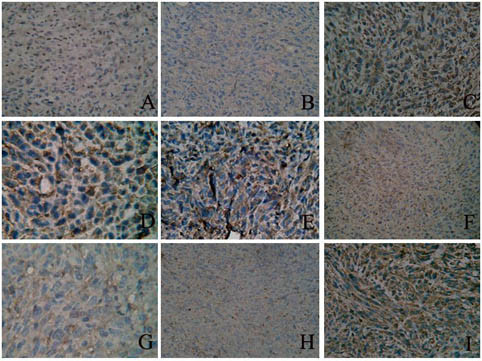
**Bax, bcl-2 and caspase-3 protein expression inthree groups cell (×400)**. (A) Bax normal SHG_44_;(B)Bax SHG_44_-EV; (C)Bax SHG_44_-DKK-1;(D) Bcl-2 normal SHG_44 _(E)Bcl-2 SHG_44_-EV; (F)Bcl-2 SHG_44_-DKK-1; (G)Caspase-3 normal SHG_44; _(H)Caspase-3 SHG_44_-EV; (I)Caspase-3 SHG_44_-DKK-1

Images of bax, caspase-3 and bcl-2 staining are shown in Figure 6. The positive reaction located in cytosol was stained in brown. The color of the stain is positively correlated to the protein expression. The IOD of each group revealed that in the SHG_44 _-DDK-1 the expression of bax and caspase-3 increased, whereas the expression of bcl-2 decreased (Table 1).

**Table 1:** Bax, bcl-2 and caspase-3 expression (in IOD) in normal SHG_44_, SHG_44_-EV and SHG_44_-DKK-1 cells.

	Bax protein expression	Bcl-2 protein express	Caspase-3 protein express
	
	n = 6 IOD	n = 6 IOD	n = 6 IOD
normal SHG_44_	2323 ± 305	5046 ± 521	1845 ± 126
SHG_44_-EV	2623 ± 420	6417 ± 462	1920 ± 231
SHG_44_-DKK-1	4567 ± 598*	2900 ± 302*	3944 ± 511*

*P < 0.05

## Discussion

The family of DKK genes is a small, but conservative gene family, which is composed of DKK-1, DKK-2, DKK-3, DKK-4 and DKKL-1 (also called Soggy), a DKK-3 related gene. DKK proteins possess different structure and function, but many of them play important roles in various human diseases [2]. DKK-1 is the most well-studied gene in the DKK gene family. It is mapped to chromosome 10q11.2 [11] and encodes a secretory glucoprotein, which contains 266 amino acids with a molecular weight of 35KD. The glucoprotein contains a N-terminal signal peptide of 31 amino acids, two conserved cysteine-rich domains and a C-terminus with glycosylation function. DKK-1 acts as a wnt antagonist by forming a complex with the transmembrane proteins Kremen1 and 2 (Krm1/2) and low- density-lipoprotein 5/6(LRP5/6). The complex is then removed through endocytosis, resulting in the removal of LRP5/6 from the cell surface [12,13] Recent studies revealed that DKK-1 is not only an antagonist of classic Wnt/β-cantenin signaling pathway but also a direct regulator of transcription of its target genes [14].

The function of DKK-1 in tumor progression has been shown to be complicated and even controversial. A number of studies showed that DKK-1 induces apoptosis and inhibits tumor growth [15-17] DKK-1 expression in primary medulloblastoma cells is significantly down-regulated relative to normal cerebellum and transfection of a DKK-1 gene construct into D283 cell line suppresses medulloblastoma tumor growth [18]. In addition, adenoviral vector-mediated expression of DKK-1 in medulloblastoma cells significantly increases the apoptosis rate. DKK-1, however, is also reported to be overexpressed in tissues and serum of lung cancers and esophageal squamous cell carcinoma, suggesting that DKK-1 may act as pro-oncogene [19]. Differential gene expression analysis showed that the resistance of the CAL27 cell line to cisplatin, an anti-cancer chemotherapy drug, is associated with the expression level of DKK-1[20]. The expression level of DKK-1, an inhibitor of canonical WNT signaling, was decreased in Cal27cis, a sub-cell line of Cal27, and was obtained by treating Cal27 with increasing concentrations of cisplatin. Overexpression of DKK-1 in both Cal27 and Cal27cis resulted in increased sensitivity to cisplatin, suggesting DKK-1 and the WNT signaling pathway as a marker and target for cisplatin chemosensitivity.

In human glioma cells, a previous study showed that transfection of DKK-1into human glioma cell line U87MG causes the cells more sensitive to cisplatin and alkylating agent [15]. Our current study revealed that the expression of bax and caspase-3 increased, whereas the expression of bcl-2 decreased in the SHG_44 _-DDK-1 cells, further confirming the pro-apoptosis function of DKK-1. We speculate that the function of DKK-1 may be tissue or cell type specific. Another possibility is that mutations of DKK-1 could be the causes of different functions of DKK-1. A screening of 73 brain tumors, however, revealed that no obvious mutations of DKK-1 were found in these brain tumors [21]. More studies, especially direct comparison of DKK-1 in different cell types at the same condition, are needed in order to better understand the complex functions of DKK-1 in relation to cancer development.

## Competing interests

The authors declare that they have no competing interests.

## Authors' contributions

YZ conceived of the study, and participated in its design and coordination and helped to draft the manuscript. WL carried out the molecular genetic studies. QX participated in its design and coordination. YH participated in the conception and the design of the analysis. All authors read and approved the final manuscript
